# Learning Beyond the Clinic: Can Point of Choice Visual Feedback Prompts Elicit Motor Behavior Changes in Persons with Multiple Sclerosis?

**DOI:** 10.21203/rs.3.rs-9348058/v1

**Published:** 2026-05-11

**Authors:** Samantha R. Fox, David C. Jangraw, Guido Mascia, Reed D. Gurchiek, Yuanyuan Feng, Brett M. Meyer, Andrew J. Solomon, Ellen W. McGinnis, Ryan S. McGinnis

**Affiliations:** University of Vermont; University of Vermont; Wake Forest University School of Medicine; Clemson University; University of Vermont; University of Vermont; The Larner College of Medicine at the University of Vermont; Wake Forest University School of Medicine; Wake Forest University School of Medicine

**Keywords:** Multiple sclerosis, fall prevention, point-of-choice intervention, visual feedback, stance width, trunk flexion, mobile health

## Abstract

**Background:**

Falls are common in persons with multiple sclerosis (PwMS), and their consequences further undermine their quality of life. Current fall prevention interventions fail to address real-time stability challenges that may occur during cognitively complex daily activities. This need may be fulfilled by means of just-in-time interventions that leverage prompts delivered via a mobile app, having the aim of producing beneficial targeted biomechanical changes.

**Methods:**

Fourteen ambulatory PwMS completed multiple Timed Up-and-Go tests under cognitive load across two different visits. Two visual feedback prompts targeting trunk flexion during sit-to-stand transitions (STS-TF) and stance width (SW) during gait were delivered terminally via ab *ad hoc* designed app, the *Just in Time Fall Prevention* app. Linear mixed-effects models with Dunnett's correction were implemented to evaluate prompt-induced changes relative to baseline.

**Results:**

Prompts were successfully able to systematically reduce STS-TF by 7.1° (p < 0.001, d = − 0.483) and to increase SW by 8.1 mm (p = 0.021, d = 0.259). The majority of participants reported willingness to use the app with and without physician prescription (95% and 68%, respectively).

**Conclusion:**

This study demonstrated for the first time that PwMS can produce targeted biomechanical adaptations in response to point-of-choice visual feedback prompts delivered by a mobile app, supporting that just-in-time mobile interventions as a proactive fall prevention strategy.

## Introduction

Falls are major contributors of several adverse outcomes in people quality of life, imposing a significant burden on the healthcare system, with annual medical costs reaching $50 billion in the US [1]. Fall risk is heightened in persons with multiple sclerosis (MS), a neuroinflammatory disease affecting nearly 3 million people worldwide [2], with more than half estimated to experience a fall in any given three-month period [3]. Fall-related injuries entail a significant long-term burden for persons with MS (PwMS), limiting mobility and independence. PwMS experience debilitating fatigue and impaired cognition, coordination, muscle strength, and sensation [4-7], collectively leading to a decline in balance and postural control [8]. Dynamic activities during daily living such as postural transfers, changes of direction, and walking pose the highest fall risk for PwMS [9, 10].

Current fall prevention interventions in MS are typically *reactive* (i.e., prescribed after a fall has been reported) rather than *proactive* [11, 12]. Reactive interventions typically include high-frequency exercise and educational programs [13-15]. While these approaches show promise for improving stability, their benefits may fade over time [16], and evidence for preventing future falls remains unclear [15, 17]. Critically, these approaches fail to address the moment-to-moment stability challenges that PwMS face during daily activities, particularly as MS-related cognitive impairment and dual-tasking demands may cause individuals to forget or fail to apply learned exercises when fall risk is highest [18]. To address this challenges, timely reminders may help prolong the beneficial effects of these interventions.

Advances in mobile health and visual feedback (VF) offer an alternative for what concerns the proactive intervention approach. VFs produced improvements in adapting immediate gait metrics for patients with other neurological diseases such as stroke [19, 20]. However, VF has only been minimally explored in PwMS [21-23]. Small studies have demonstrated improvements in gait speed and stride length from VF via virtual reality headsets [24] and cycling VF training [25], and balance improvements after Nintendo Wii Fit training [26]. However, several limitations emerge; first, it remains unclear whether PwMS can effectively react to specific visual instructions aimed at producing targeted biomechanical changes. Secondly, the proposed approaches require cumbersome equipment, making the delivery of the associated VFs outside of the laboratory problematic, jeopardizing their ecological validity. Critically, although VFs showed promise in inducing beneficial biomechanical changes, they require constant vigilance aimed at preserving them [22].

Recent biomechanical research has identified specific movement patterns associated with fall risk in PwMS, with fallers exhibiting reduced static and dynamic balance [27]. Specifically, the dynamic margin of stability (MoS) has emerged as a promising measure given its direct relationship to dynamic stability and fall risk [28, 29]. The two key biomechanical quantities that directly influence MoS are stance width (SW) during walking, which defines the size of the base of support (BoS) [30, 31], and trunk flexion during sit-to-stand transitions (STS-TF), which affects center of mass (CoM) control as the individual performs a postural transfer from a static position to a dynamic movement [32]. However, the ability to modify these biomechanical quantities through targeted VF interventions has not been systematically investigated in PwMS. Nonetheless, mobile apps offer a viable, ecologically valid opportunity to administer VFs promptly, ubiquitously and on a scale. However, to the best of our knowledge, no study evaluated the efficacy of VFs delivered via a mobile app in proactively modifying biomechanical quantities of interest in PwMS.

Thus, the primary objective of this work is to investigate if VF prompts delivered via a mock-mobile app, called *Just in Time Fall Prevention* (JiTFP), can induce immediate, targeted biomechanical changes improving stability in PwMS. We evaluate SW and STS-TF responses leveraging Timed Up-and Go (TUG) tests performed under cognitive load to mimic real-life dual-tasking conditions [33, 34]. Finally, we examined the participants’ willingness to use the JiTFP app to help manage their fall risk with and without their doctor actively prescribing its use.

## Methods

### Study design

The initial sample [35] included 22 ambulatory PwMS with no cause of mobility impairment other than MS. This study was approved by the University of Vermont Institutional Review Board (CHRMS 22-0401). Informed consent was obtained from all participants included in the study. All the procedures in this study were performed in accordance with the Declaration of Helsinki.

The study involved data from two phases: i) an assessment visit (AV), where the participants’ baseline biomechanics were assessed; ii) a point-of-choice prompt visit (PV), where VF prompts were delivered via the JiTFP app, and their biomechanical effects subsequently measured. COVID severely impacted the recruitment, hence the PV phase occurred either immediately after or up to 7 months later.Seventeen participants completed both phases, three of which were later excluded due to data loss. The final sample included 14 participants (11 Female; 6 fallers; mean age ± standard deviation 48.1 ± 9.2 years). In the AV phase, participants’ fall status was determined via self-reported fall history within the previous six months using the Falls, Trips, and Slips survey [36],and disease status was determined via Patient-Reported Expanded Disability Status Scale [37] (PR-EDSS; mean score ± standard deviation 3.7 ± 0.59). Finally, at the end of the PV phase, participants completed a four-item questionnaire designed to probe their willingness to use the JiTFP app and their trust in its recommendations.

#### Prompt Development

The prompts were designed to use evidence-based strategies for reducing fall risk commonly recommended during physical therapy [38], combined with principles from behavioral psychology and motor learning theory. Specifically, prompts were developed based on point-of-choice (POC) intervention principles, known to affect behavior change in healthy populations [39-41].

**Figure 1:**
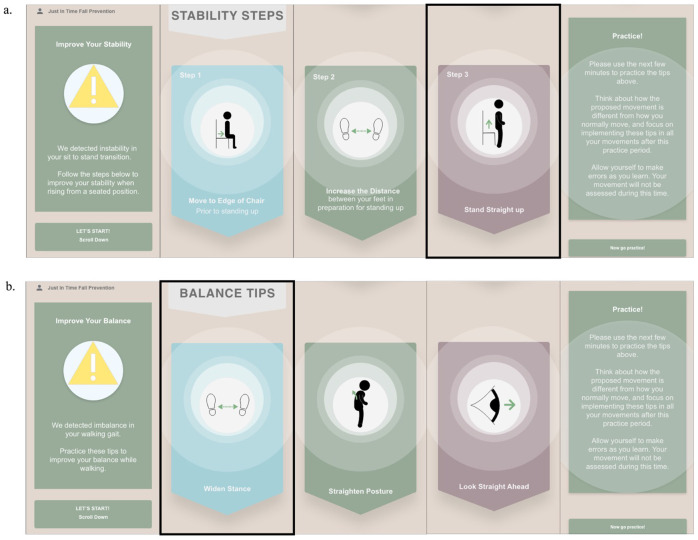
Visualization of the Just in Time Fall Prevention app prompts for Sit-to-Stand (a.) and Gait (b.) improvement. In this study, only responses to step 3 and step 1 were analyzed for Sit-to-Stand and Gait, respectively, here highlighted by the black rectangles.

We designed two prompts, each composed of three steps (Figure 1), affecting STS and gait, respectively:

- **STS prompt**: designed to reduce STS-TF in the sagittal plane, prompting a more upright posture that maintains the CoM within the BoS [42]. In this study, we analyzed responses to the trunk posture component (Figure 1a, step 3), which promotes reduced STS-TF, consequently improving transfer stability [42].
- **Gait prompt**: designed to increase SW during walking phases, as voluntary increases in SW enhance dynamic MoS [31]. In this study, we specifically analyzed responses to the stance-widening component of the prompt (Figure 1b, step 1), which directly targets lateral stability by expanding the BoS in the medial-lateral direction.

Prompt design combined text and graphics (Figure 1) to reduce cognitive load through distributed information processing [43].

### Experimental Protocol

#### Assessment Visit (AV)

The AV phase was conducted in the M-Sense Research Laboratory at the University of Vermont. Following written informed consent, participants were instrumented with a custom reflective marker set based on standard placement protocols for trunk and lower-body biomechanical analysis [44]. Three-dimensional motion capture data were collected using a 19-camera system (Vicon Motion Systems Ltd, Oxford, UK) sampling at 100 Hz.

**Figure 2:**
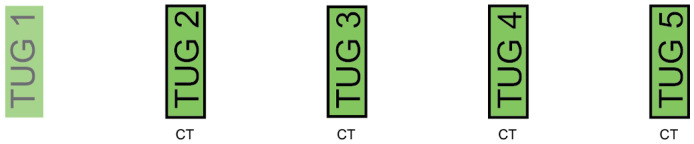
Schematic depiction of the Assessment Visit (AV) protocol. Data from outlined boxes was included in the analysis described in the Data Processing section; data from shaded, borderless boxes was not included in the analysis. Legend: TUG = Timed-Up and Go test; CT = cognitive task, indicating that activity was performed with concurrent cognitive load.

Participants performed supervised tasks as part of the larger study [35]. In the following analysis, we consider data measured during TUGs (Figure 2). Participants performed 5 standard TUGs, except for the use of an armless, backless chair to facilitate tracking of optical motion capture markers [45]. The first TUG served as a familiarization trial to acclimate participants to the task and was not included in the analysis described below. The subsequent 4 TUGs incorporated concurrent cognitive load to induce realistic dual-tasking demands commonly encountered in daily life[33,34]. Cognitive tasks included categorical verbal fluency tasks (“Name as many countries/cities/car models/animals as possible”) as they performed each TUG, and were administered in the same order for all participants.

#### Point-of-Choice Visit (PV)

The PV phase was designed to evaluate the efficacy of VF prompts in altering biomechanical patterns. Two distinct VF prompts were delivered via the JiTFP app (Figure 1).

**Figure 3:**
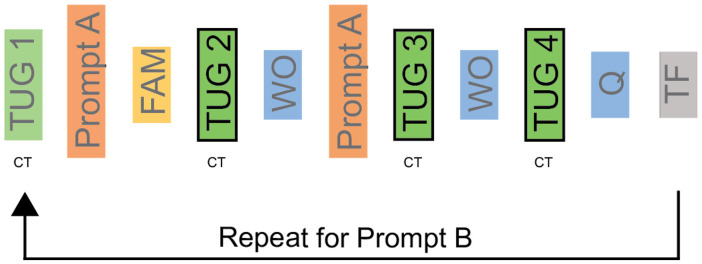
Schematic depiction of the Point-of-Choice Visit (PV) feedback protocol. Data from outlined boxes was included in the analysis described in the Data Processing section; data from shaded, borderless boxes was part of the PV protocol, but it was not included in the analysis. Legend: TUG = Timed-Up and Go test; Prompt A = First prompt shown in the app; FAM = Familiarization period; WO = Wash-out period; Q = Questionnaire; TF = Transfer test; CT = cognitive task, indicating that activity was performed with concurrent cognitive load.

Participants performed four TUGs during the PV phase following a structured protocol incorporating washout periods to minimize carryover effects and familiarization periods to practice the prompts (Figure 3). The first TUG occurred before the prompts and was not included for further analysis. In this specific protocol, prompt order was randomized to control for sequence effects. Participants were shown each prompt on a mobile phone twice, regardless of their stability. Prompt A refers to the one randomly provided to the participant first. Prompts were provided after a TUG was performed. A familiarization period (FAM) occurred after the first time a participant saw each prompt, allowing participants to practice them. Washout periods included activities unrelated to the study or prompt (e.g., walking down the hallway, having a conversation, completing a questionnaire). The feasibility questionnaire was administered last.. Washout periods took 3-5 minutes. Each TUG was performed under the same AV dual-task conditions to retain ecological validity. Moreover, transfer tests were implemented to evaluate if learned behaviors transferred to other activities (not included in this analysis).

### Data Processing

Motion capture data were processed using Vicon Nexus (v.2.12.1) to calibrate marker positions into a body-aligned coordinate frame with a static calibration trial.

Marker trajectories were low-pass-filtered (4th-order Butterworth filter; cutoff frequency = 6 Hz), and *ad hoc* algorithms, were designed to detect the movement phases sit-to-stands and gait phases during TUGs, subsequently analyzed to extract biomechanical measures.

Sit-to-stands were identified using the vertical displacement of the sacrum marker, with the movement initiation being detected at the instant when the vertical velocity exceeded two standard deviations above baseline. The sit-to-stand phase was defined as beginning 0.5 seconds prior to detected upward movement and ending when the sacrum marker reached the maximum vertical displacement (full upright posture).

The two gait phases in the TUG were separated by the 180-degree turn, identified as the point of maximum anterior-posterior displacement of the sacrum marker relative to its location at the beginning of the task. To isolate gait from turning and sitting, two steps before and two steps after the turn were excluded from analysis while the first step after standing was included. The first gait phase extended from sit-to-stand completion to two steps before the turn, while the second gait phase began two steps post-turn and concluded when anterior-posterior and vertical sacrum trajectories crossed, indicating movement termination (final stand-to-sit).

Two biomechanical metrics were calculated based on their established relationship to MoS and fall risk reduction: i) the STS-TF angle, evaluating the STS prompt effectiveness in promoting an upright posture during transfers, helping maintain the CoM within the BoS; ii) the SW during steady-state gait, assessing the gait prompt’s ability to increase SW, improving MoS by increasing the BoS. Specifically, STS-TF angle was computed as the maximum angle between the long axis of the trunk and the vertical axis in the world frame during an sit-to-stand, whereas SW was computed as the medial-lateral distance between right and left heel markers during each of the gait phases. Both metrics were averaged across their respective phases within each TUG to provide representative values for statistical analysis.

### Statistical Analysis

To evaluate prompt-induced biomechanical changes, and to account for repeated measures, we employed a within-subjects linear mixed-effects model (LMM) for each biomechanical metric (maximum STS-TF angle and SW during steady-state gait), with three experimental conditions: Control (AV baseline), Gait Prompt, and STS Prompt. Conditions were included as fixed effects, while participant IDs were treated as random effects to account for individual variability. Control TUGs from the AV phase served as the baseline comparison for both metrics.

Multiple comparisons were adjusted via Dunnett's test [46] to compare the experimental conditions against the control condition. Effect sizes were calculated using Cohen's d to quantify the magnitude of observed changes. All analyses were conducted using Python (v3.9.12).

## Results

LMMs were fit to analyze the effects of two prompt conditions (Gait Prompt and STS Prompt) compared to control on biomechanical outcomes during TUGs. Each of the 14 subjects had 10 TUGs (4 Control, 3 Gait Prompt, 3 STS Prompt) that were included in the analysis.

**Figure 4:**
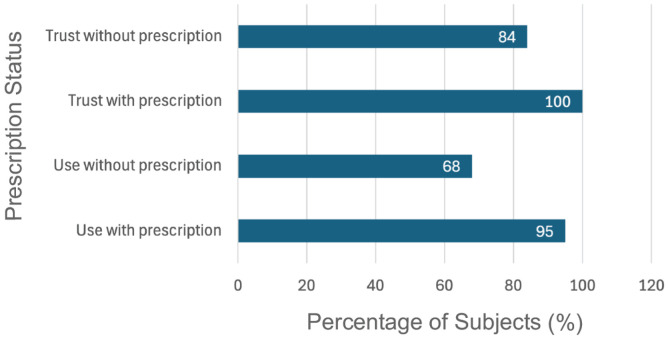
Just in Time Fall Prevention Application Acceptance and Trust by Prescription Status. The Prescription Status indicates whether their doctor were to prescribe the use of the app or not.

After correction for multiple comparisons, the STS Prompt yielded a significant reduction in STS-TF angle compared to control by −7.1 degrees (p < 0.001, d = −0.483, Table 1). Similarly, the Gait Prompt significantly increased SW by 8.1mm compared to control (p = 0.021, d = 0.259, Table 2). Finally, the analysis from the post-intervention questionnaire for the JiTFP app (Fig. 4) revealed high trust (84–100%) and willingness of use (68–95%).

## Discussion

This study investigated whether PwMS can produce targeted biomechanical changes in response to specific VF prompts delivered via a realistic mock mobile application. Our results demonstrate that PwMS can modify specific movement patterns in response to targeted visual instructions: the STS Prompt significantly reduced STS-TF angle by 7.1° during sit-to-stands (Table 1), while the Gait Prompt significantly increased SW by 8.1mm during steady-state walking (Table 2). Additionally, survey results (Fig. 4) indicated that most participants would be interested in using the app to help manage their fall risk (95% with prescription, 68% without) and would trust the app’s recommendations (100% with prescription, 84% without).

The 8.1mm increase in SW observed in response to the Gait Prompt extends recent findings that PwMS can immediately adapt their gait to external feedback [47]. Due to sensory impairments commonly observed in PwMS [48], researchers have utilized various forms of external feedback to enhance sensory input and improve motor control, including textured insoles [47] and, to a limited extent, VF approaches [24, 25]. Previous studies utilizing VF demonstrated improvements in gait speed and stride length through virtual reality training [24], and VF during cycling [25]. Our findings suggest that responses to external feedback with an internal focus on the participant's own body movements ("widen your stance", Fig. 1b, step 1) may be beneficial, in contrast to the external focus on external targets or gaming elements employed in previous VF studies [24, 25, 47]. This distinction is crucial, since the constrained action hypothesis suggests that focusing internally on movement constrains automatic control processes, while external focus leverages these processes [49]. Shafizadeh et al. [50] assessed both approaches in PwMS, finding that external focus improved gait speed and stride length more effectively than internal focus, supporting the hypothesis despite deteriorated neuromotor pathways in MS. However, a critical distinction exists between their work and ours: previous studies targeted optimization of natural movement parameters (speed, stride length) that may align with what automatic movement regulation systems inherently attempt to achieve. In contrast, our study instructed participants to perform specific compensatory behaviors that may feel unnatural and may not be part of their natural automatic movement patterns. Thus, to encourage such deliberate compensatory strategies, we chose an internally focused prompt which may be necessary to override automatic processes and consciously implement protective behaviors that the impaired neuromotor system might not naturally execute.

Moreover, PwMS have been observed to utilize a wider SW as a compensatory strategy to enhance lateral stability during slower walking [29], which may explain the small effect size observed (d = 0.259), as an already-widened stance limits further improvement. Alternatively, this may be due to variable MS symptoms such as increased spasticity, limiting lower extremity abduction [51]. Nevertheless, the ability to voluntarily enhance this mechanism during dual-tasking is significant, because falls often occur during cognitively demanding activities [52]. Indeed, the proactive capacity to respond to visual prompts while managing concurrent cognitive demands may suggest that, while stance widening may be part of their compensatory repertoire, conscious attention to this strategy can optimize its implementation. This supports the utility of POC feedback as real-time reminders to consciously engage protective behaviors when needed most.

PwMS often exhibit increased STS-TF as a compensatory strategy to overcome lower limb weakness [53]. While this strategy uses momentum to aid upward movement, it shifts the CoM outside the BoS, creating instability [53]. The 7.1° reduction in trunk flexion angle demonstrates that PwMS can successfully modify this postural control strategy through VF. This conscious modification of an existing compensatory pattern may require internal focus to override automatic movement processes that have adapted to use trunk flexion as a primary strategy for sit-to-stands. The medium effect size (d=−0.483) suggests moderate modification and likely reflects the different intervention approach: modifying a problematic compensatory behavior (STS-TF) versus enhancing a beneficial one (SW). Targeting reduction of an inefficient movement pattern provides greater room for improvement compared to further enhancing an already present adaptation, consistent with previous research indicating that increased impairment is correlated with increased adaptations [54].

The high acceptance rates (Fig. 4) provide encouraging evidence for clinical translation potential. Survey results show that 95% of participants would use the application if prescribed by their doctor, and 68% would use it even without physician prescription. Notably, over 25% attributed wanting to use the app specifically for its reminders, suggesting that participants recognize appropriate behaviors and value proactively prompted reminders. This aligns with POC intervention principles, addressing a critical gap: while traditional fall prevention interventions show diminishing effects over time [21], reminder-based interventions could provide sustained benefits.

This study demonstrates the potential of terminal VF for eliciting biomechanical changes in PwMS. However, selection of feedback modality requires careful consideration for future deployment of this tool. PwMS rely heavily on vision for balance [55], making concurrent visual prompts potentially counterproductive during walking. Alternative modalities such as vibrotactile or auditory feedback linked to learned visual cues [43] may be more appropriate for real-time intervention. Given the reduced attentional capacity observed in PwMS, any concurrent feedback must be carefully designed to avoid increasing fall risk. Similarly, personalization based on individual fall-risk profiles will likely be essential for clinical implementation. Future studies should assess whether these biomechanical modifications translate to improved stability and reduced fall occurrence in daily life, particularly in individuals exhibiting specific deficiencies in SW or TF control.

Despite the important preliminary findings of this work, several limitations warrant consideration. First, sample size was small and included PwMS with limited disability (per PR-EDSS), yielding insufficient power to examine whether prompt responses differed by age, sex, or disability severity. Second, dual-tasking costs were not calculated in this study, leaving uncertainty about attention allocation during real-world distractions. Future implementations should evaluate whether participants can achieve desired biomechanics while managing real-life environmental demands. Third, prompts were provided regardless of current stability status. While research has supported the ability of PwMS to respond to real-time perturbations [56], it remains unknown whether PwMS can adapt their biomechanics in response to VF while experiencing instability. Since falls typically result from consecutive unstable steps rather than a single unstable step [57], POC interventions may provide opportunities to interrupt this cascade. Future research should evaluate optimal feedback timing relative to existing instability.

## Conclusion

This study provides initial evidence that PwMS can produce immediate, targeted biomechanical adaptations in response to POC VF prompts delivered via a mobile application. Participants modified motor behavior associated with improved dynamic stability including reduced STS-TF and increased SW during gait. Importantly, survey results showed high user acceptance of the intervention app. These findings support the feasibility of leveraging just-in-time, prompt-based interventions to translate motor strategies into cognitively demanding, real-world conditions. Future research should optimize feedback modalities and timing and determine whether these biomechanical changes translate to meaningful reductions in fall risk.

## Figures and Tables

**Table 1:** Sit-to-Stand Trunk Flexion LMM. SE = Standard Error; CI = Confidence Interval. Statistically significant results are indicated in bold.

Condition	Estimate (°)	SE	z	95% CI	p- value	Adjusted p- value	Cohen’s D
Intercept	73.833	4.029	18.326	[69.9, 81.7]	< 0.001	-	-
Gait Prompt (vs Control)	−2.330	1.720	−1.355	[−5.7, 1.0]	0.176	0.356	-
STS Prompt (vs Control)	−7.050	1.720	−4.099	[−10.4, −3.7]	< 0.001	**< 0.001**	−0.483

**Table 2:** Gait Stance Width LMM. SE = Standard Error; CI = Confidence Interval. Statistically significant results are indicated in bold.

Condition	Estimate (mm)	SE	z	95% CI	p- value	Adjusted p- value	Cohen’s D
Intercept	126.821	6.932	18.294	[113.2, 140.4]	< 0.001	-	-
Gait Prompt (vs Control)	8.059	3.096	2.603	[2.0, 14.1]	0.009	**0.021**	0.259
STS Prompt (vs Control)	5.339	3.096	1.725	[−0.7, 11.4]	0.085	0.174	-
